# Gene Expression Profiling Combined with Bioinformatics Analysis Identify Biomarkers for Parkinson Disease

**DOI:** 10.1371/journal.pone.0052319

**Published:** 2012-12-28

**Authors:** Hongyu Diao, Xinxing Li, Sheng Hu, Yunhui Liu

**Affiliations:** 1 Department of Neurosurgery, Shengjing Hospital of China Medical University, Shenyang, China; 2 Department of Neurosurgery, The Second People’s Hospital of Chaoyang City, Chaoyang, China; Emory University, United States of America

## Abstract

Parkinson disease (PD) progresses relentlessly and affects approximately 4% of the population aged over 80 years old. It is difficult to diagnose in its early stages. The purpose of our study is to identify molecular biomarkers for PD initiation using a computational bioinformatics analysis of gene expression. We downloaded the gene expression profile of PD from Gene Expression Omnibus and identified differentially coexpressed genes (DCGs) and dysfunctional pathways in PD patients compared to controls. Besides, we built a regulatory network by mapping the DCGs to known regulatory data between transcription factors (TFs) and target genes and calculated the regulatory impact factor of each transcription factor. As the results, a total of 1004 genes associated with PD initiation were identified. Pathway enrichment of these genes suggests that biological processes of protein turnover were impaired in PD. In the regulatory network, HLF, E2F1 and STAT4 were found have altered expression levels in PD patients. The expression levels of other transcription factors, NKX3-1, TAL1, RFX1 and EGR3, were not found altered. However, they regulated differentially expressed genes. In conclusion, we suggest that HLF, E2F1 and STAT4 may be used as molecular biomarkers for PD; however, more work is needed to validate our result.

## Introduction

Parkinson disease (PD) is a common chronic neurodegenerative disorder characterized by selective loss of dopaminergic neurons from the substantia nigra and presence of Lewy bodies [1]. The obvious symptoms are tremor at rest, muscle rigidity, bradykinesia and other movement-related symptoms [2]. PD is difficult to diagnose in its early stages, and when it was diagnosed, the only treatment involved boosting inadequate levels of dopamine in the brain, which did not eliminate all symptoms. Therefore, it is of significantly importance to find molecular biomarkers of PD to improve diagnosis accuracy, monitor disease progression and develop therapeutic interventions.

The etiology of PD remains a puzzling mix of environmental factors, genes and the aged brain [3], [4]. Epidemiological research indicates that exposure to pesticides elevates the risk of PD. By contrast, caffeine and tobacco are associated with reduced risk [5]. In recent years, several causative genes of PD have been identified, including α-synuclein (SNCA), parkin (PARK2), UCHL-1 (PARK5), PINK1 (PARK6), DJ-1 (PARK7), LRRK2 (PARK8) and ATP13A2 (PARK9) [6], [7]. These PD-linked molecules are candidate biomarkers for PD [8]. Among them, the levels of DJ-1 and α-synuclein in human cerebrospinal fluid and blood between PD patients and non-PD controls are the most frequently tested biomarkers in previous studies; however, the results are conflicting [9], [10], [11], [12], [13]. At this stage, neither DJ-1 nor α-synuclein alone appears to be satisfactory as the biological biomarker for PD. Besides, changed levels of Urinary 8-hydroxydeoxyguanosin (Urinary 8-OHdGe) and proinflammatory cytokines such as tumor necrosis factor α (TNF-α), interleukin 6 (IL-6) and interleukin 1β (IL-1β) are also been studied as biomarkers for PD [14], [15]. Godau et al. recently showed that the levels of serum insulin-like growth factor (IGF-2) were significantly higher in PD patients than that in controls [16].

The purpose of this study is to identify molecular biomarkers for PD initiation using a computational bioinformatics analysis of gene expression. The availability and integration of high-throughput gene expression data and the computational bioinformatics analysis may shed new lights on molecular biomarker identification of PD.

## Materials and Methods

### Affymetrix Microarray Data

The transcription profile of GSE 20333 was downloaded from a public functional genomics data repository GEO (Gene Expression Omnibus) (http://www.ncbi.nlm.nih.gov/geo/). Affymetrix HG-Focus array was used to determine a global gene expression profile of clinically and neuropathologically confirmed cases of sporadic Parkinson disease (*n* = 6) compared to controls (*n* = 6). Postmortem human brains were obtained from moderately to severe Parkinsonism individuals based on the Hoehn & Yahr criteria. The average age for PD and control is 76.6 and 77.8 years, respectively. The average postmortem delay for PD and control is 26.2 and 19.8 hours, respectively.

### Pathway Data

KEGG (Kyoto Encyclopedia of Genes and Genomes) is one of the most popular pathway databases; it groups genes into pathways of interacting genes and substrates, and contains specific links between genes and substrates that interact directly [17], [18]. The PATHWAY database records networks of molecular interactions in the cells, and variants of them specific to particular organisms (http://www.genome.jp/kegg/). We collected pathway information from KEGG on June 30, 2011.

### Regulatory Data

UCSC (http://genome.ucsc.edu) is an interactive website offering access to genome sequence data from a variety of vertebrate and invertebrate species and major model organisms, integrated with a large collection of aligned annotations. We downloaded the human transcription factors (TFs) and their target chromosome region from UCSC. Then, we downloaded the chromosome annotation information from NCBI and analyzed the relationships between TFs and their target genes.

### Differentially Coexpression Analysis

From the perspective of systems biology, functionally related genes are frequently coexpressed across a set of samples [19], [20], [21]. Differentially Coexpressed Genes and Links (DCGL) is designed for identifying differentially coexpressed genes and links from gene expression microarray data [22].

For GSE20333, we used the DCGL package [22], [23] in R [24] to identify differentially coexpressed genes (DCGs) and links in PD patients compared to non-PD controls. We calculated the *p*-values and adjusted the raw *p*-values into false discovery rate (FDR) using the Benjamini-Hochberg method [25] to circumvent the multi-test problem which might induce too much false positive results. The genes only with FDR <0.25 were selected as differentially coexpressed genes.

### Pathway Enrichment Analysis

In order to facilitate the functional annotation and analysis of large lists of genes in our result, we inputted all the DCGs into DAVID (The Database for Annotation, Visualization and Integrated Discovery) for KEGG (Kyoto Encyclopedia of Genes and Genomes) term enrichment analysis. The DAVID identifies canonical pathways associated with a given list of genes by calculating the hypergeometric test p-value for probability that association between this set of genes and a canonical pathway [26]. We chose *p*-value <0.05 as the cut-off criterion.

### Measures of RIF

Regulatory impact factor (RIF) appears to be a robust and valuable methodology to identify the regulators with the highest evidence of contributing to differential expression in two biological conditions. It is a metric given to each TF that combines the change in coexpression between the TF and the DEGs (i.e. the potential targets). The measures of RIF are computed as follows [27]:
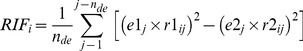
(1)where *n_de_* is the number of DEGs; *e*1*_j_* and *e*2*j* represent the expression value of the *j*th DEG in conditions 1 and 2, respectively; *r*1*_ij_* and *r*2*_ij_* represent the coexpression correlation between the *i*th TF and the *j*th DEG in conditions 1 and 2, respectively.

## Results

### Identification of Differentially Coexpressed Genes in PD

We downloaded publicly available microarray dataset GSE20333 from GEO database and applied DCGL package in R to identify DCGs in 6 PD patients and 6 non-PD controls. Among all genes tested, we found a total of 1004 DCGs with FDR <0.25. Besides, a total of 459683 links were predicted among these DCGs.

### Enrichment of PD Associated Pathways

In order to functional annotation of the large lists of genes in our result, we used the online biological classification tool DAVID and observed significant enrichment of these genes in multiple KEGG categories (Table 1). Pathway analysis revealed that the DCGs were strongly associated with Ribosome (*p* = 2.21E-06), and Neurotrophin signaling pathway (*p* = 1.45E-04). In addition, Steroid biosynthesis, Spliceosome, and NOD-like receptor signaling pathway showed evidence of association with the differentially co-expressed genes (*p*<0.01).

**Table 1:** The enriched KEGG pathways (p<0.05).

ID	P-value	Count	Size	Term
3010	2.21E-06	13	88	Ribosome
100	0.001496	4	17	Steroid biosynthesis
3040	0.001795	11	128	Spliceosome
4621	0.002702	7	62	NOD-like receptor signaling pathway
4610	0.018662	6	69	Complement and coagulation cascades
5215	0.019005	7	89	Prostate cancer
980	0.021211	6	71	Metabolism of xenobiotics by cytochrome P450
982	0.023986	6	73	Drug metabolism - cytochrome P450
140	0.027895	5	56	Steroid hormone biosynthesis
72	0.029307	2	9	Synthesis and degradation of ketone bodies
4612	0.031962	6	78	Antigen processing and presentation
620	0.033264	4	40	Pyruvate metabolism
5210	0.040861	5	62	Colorectal cancer
4962	0.044997	4	44	Vasopressin-regulated water reabsorption
30	0.04856	3	27	Pentose phosphate pathway

* ID represents the pathway ID in KEGG. Count represents the number of DCGs enriched in each pathway. Size represents the total number of genes in each pathway. Term represents the pathway name.

### Regulatory Network Construction

**Figure 1:**
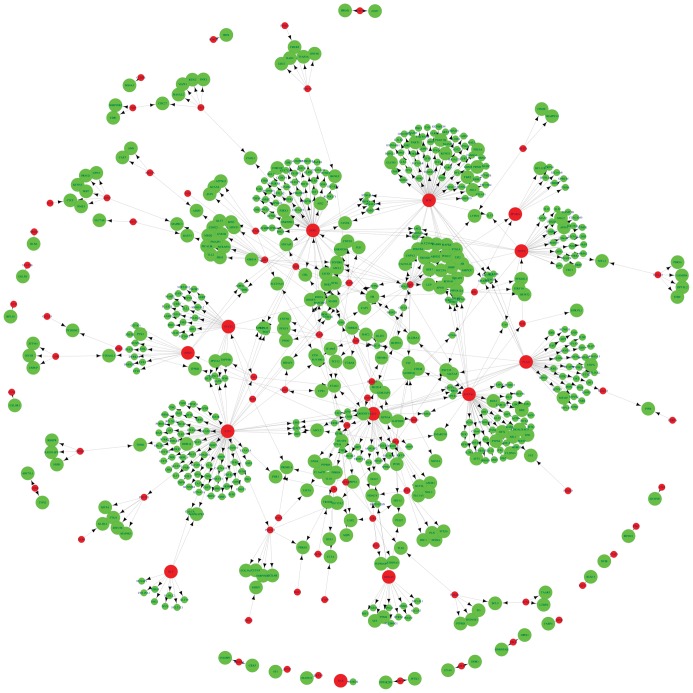
Regulatory network construction among TFs and their target genes. The red nodes represent TFs and the green nodes represent their target genes. Large nodes are differentially co-expressed genes and small nodes are non-DCGs.

We matched the 1004 DCGs and the 459683 links to the known regulatory data between transcription factors (TFs) and target genes, and obtained a total of 745 pairs of relationships between 82 TFs and 601 target genes. By integrating the regulatory relationships above, we built a regulatory network using Cytoscape [28] (Figure 1).

### Impact Analysis of Transcription Factor

**Figure 2:**
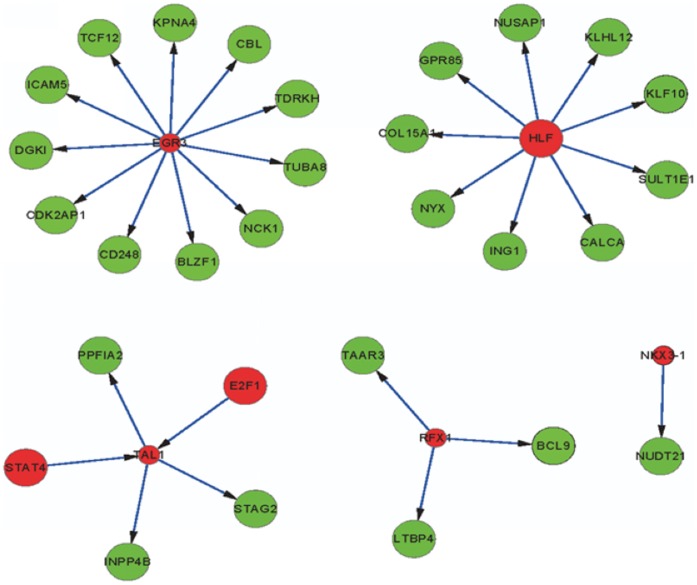
The regulatory relationships between the top 5 TFs and their target genes. The red nodes represent transcription factors and the green nodes represent their target genes.

The above network generates vast amounts of data. In order to focus on the most meaningful information, we calculated the RIF of each TF. The top 5 ranked TFs are HLF (hepatic leukemia factor), NKX3-1 (NK3 homeobox 1), TAL1 (T cell acute lymphocytic leukemia 1), RFX1 (regulatory factor X, 1) and EGR3 (early growth response 3) (Table 2). The relationships between these top 5 TFs and their target genes were shown in Figure 2 and Table 3. From Table 3, we could find that HLF, E2F1 (E2F transcription factor 1) and STAT4 (signal transducer and activator of transcription 4) are both TFs and DCGs. Other TFs, such as NKX3-1, TAL1, RFX1 and EGR3, are not DCGs, but their target genes are.

**Table 2:** The top 5 ranked TFs.

TF	RIF	Rank
HLF	121368.2	1
NKX3-1	112874.1	2
TAL1	109026.6	3
RFX1	103119.9	4
EGR3	102361.6	5

* TF represents the transcription factor. RIF represents regulatory impact factor of TF. Rank represents the impact rank of TF.

**Table 3:** The regulatory relationships between the top 5 TFs and their target genes.

TF	TargetGene	cor.1	cor.2	DCG
HLF	GPR85	0.970975	−0.88501	HLF
HLF	ING1	0.563873	−0.93968	HLF
HLF	KLHL12	−0.03165	−0.99013	HLF
HLF	NUSAP1	0.227138	−0.96687	HLF
HLF	NYX	0.294478	−0.95254	HLF
HLF	SULT1E1	0.963595	−0.28899	HLF
HLF	CALCA	−0.03387	−0.91528	HLF
HLF	COL15A1	−0.053	−0.85312	HLF
HLF	KLF10	−0.01067	−0.90286	HLF
NKX3-1	NUDT21	−0.39249	0.968038	NUDT21
TAL1	INPP4B	−0.5512	0.962619	INPP4B
TAL1	PPFIA2	−0.12643	0.964919	PPFIA2
TAL1	STAG2	−0.08273	−0.93227	STAG2
E2F1	TAL1	−0.63941	0.995085	E2F1
STAT4	TAL1	−0.54116	0.964951	STAT4
RFX1	TAAR3	−0.91249	0.91653	TAAR3
RFX1	BCL9	0.046861	0.870466	BCL9
RFX1	LTBP4	−0.28708	0.991056	LTBP4
EGR3	CBL	−0.13715	0.969967	CBL
EGR3	CD248	−0.47312	0.983806	CD248
EGR3	CDK2AP1	0.810745	−0.91525	CDK2AP1
EGR3	DGKI	−0.25926	0.95291	DGKI
EGR3	ICAM5	0.025647	0.824395	ICAM5
EGR3	KPNA4	−0.04424	0.970174	KPNA4
EGR3	NCK1	−0.69186	0.954629	NCK1
EGR3	TCF12	0.835666	−0.89573	TCF12
EGR3	TUBA8	−0.10359	0.981482	TUBA8
EGR3	BLZF1	−0.19994	0.959835	BLZF1
EGR3	TDRKH	−0.9193	0.793759	TDRKH

* TF represents the transcription factor. Target gene represents the target gene of transcription factor. cor.1 and cor.2 represent the coexpression correlation between the TF and the target gene in conditions 1 and 2, respectively. DCG indicates the differentially co-expressed gene of a pair of TF and target gene.

## Discussion

Molecular biomarkers are useful to improve diagnosis, to predict clinical behavior and to demonstrate new therapeutic efficacy. Since microarray can interrogate expression levels of thousands of genes in human genome simultaneously, it has been widely used in discovery of disease biomarkers [29], [30], [31]. In this work, we have analysed gene expression data with computational methods with the aim of uncovering genes that potentially dysregulate in PD. We identified a total of 1004 DCGs in PD patients compared to non-PD controls. After regulatory network construction and regulatory impact factor analysis, we found that the transcription factors HLF, E2F1, STAT4, NKX3-1, TAL1, RFX1 and EGR3 may play important roles in PD initiation. Of these, HLF, STAT4 and E2F1 were found have altered expression levels in PD patients. The expression levels of other transcription factors, NKX3-1, TAL1, RFX1 and EGR3, were not found altered. However, they regulated differentially expressed genes.

HLF encodes a member of the proline and acidic-rihc protein family, a subset of the bZIP transcription factors. Chromosomal translocations fusing portions of this gene with the E2A gene cause a subset of childhood B-lineage acute lymphoid leukemias [32]. While HLF has been linked to malignancies of the lymphoid system, it is detected in the liver, kidney, and adult nervous system by northern blotting [33]. Hitzler et al. found that HLF expression increased markedly with synaptogenesis and was coincident with barrel formation and suggested that HLF plays a role in the function of differentiated neurons in the adult nervous system [34]. HLF appears as the most significant transcription factors related to the differential expression of genes in PD patients.

E2F1 is a member of the E2F family of transcription factors. The E2F family plays a crucial role in the control of cell cycle and action of tumor suppressor proteins and is also a target of the transforming proteins of small DNA tumor viruses. Several studies have demonstrated that E2F1 contributes to neuronal damage and death using in vitro models of neurodegeneration [35], [36], [37]. E2F1 immunoreactivity and/or protein levels were reported to increase in neurons of patients with PD [38]. They showed that pRb/E2F pathway is activated in dopaminergic neurons in PD, but also demonstrated that activation of this pathway is instrumental in the degeneration of these neurons in the MPTP/MPP+ model of the disease [38]. In a recent study, Lu and his colleagues showed that mutations in LRRK2 cause PD through inhibiting the translational repression of the transcription factors E2F1 and DP [39].

STAT4 is a transcription factor belonging to the signal transducer and activator of transcription protein family [40]. STAT4 is involved in the signaling of interleukin-12 and interferon -γ, as well as interleukin-23 [41]. Though we found STAT4 was differentially expressed in PD patients compared to non-PD controls, the gene has no known role in PD pathogenesis to data.

From Table 1, we could find that the most significant enriched pathway is ribosome which is responsible for catalyzing the formation of proteins from individual amino acids. Besides, some pathways associated with protein synthesis were also enriched in the result, such as ribosome, steroid biosynthesis, and spliceosome. This result suggests that biological processes of protein turnover were impaired in PD. Our result is in line with previous study [42], [43].

In conclusion, we have identified molecular biomarkers for PD initiation using a computational bioinformatics analysis of gene expression. A total of 1004 differentially coexpressed genes were identified between PD patients and non-PD controls. Pathway enrichment of these genes suggests that biological processes of protein turnover were impaired in PD. After regulatory network construction and regulatory impact factor analysis, we found that the transcription factors HLF, E2F1, STAT4, NKX3-1, TAL1, RFX1 and EGR3 may play important roles in PD initiation. Of these, HLF, STAT4 and E2F1 were found have altered expression levels in PD patients. Therefore, we suggested that HLF, E2F1 and STAT4 may be used as biomarkers for PD; however, more work is needed to validate our result.
